# Pneumococcal vaccination and otitis media in Australian Aboriginal infants: comparison of two birth cohorts before and after introduction of vaccination

**DOI:** 10.1186/1471-2431-9-14

**Published:** 2009-02-19

**Authors:** Grant Austin Mackenzie, Jonathan Rhys Carapetis, Amanda Jane Leach, Peter Stanley Morris

**Affiliations:** 1 Bacterial Diseases Program, Medical Research Council (UK) Laboratories, Fajara, Gambia; 2 Child Health Division, Menzies School of Health Research, Darwin, NT, Australia; 3 Institute for Advanced Studies, Charles Darwin University, Darwin, NT, Australia; 4 School of Medicine, Flinders University of South Australia, Adelaide, SA, Australia; 5 NT Clinical School, Flinders University of South Australia, Darwin, NT, Australia

## Abstract

**Background:**

Aboriginal children in remote Australia have high rates of complicated middle ear disease associated with Streptococcus pneumoniae and other pathogens. We assessed the effectiveness of pneumococcal vaccination for prevention of otitis media in this setting.

**Methods:**

We compared two birth cohorts, one enrolled before (1996–2001), and the second enrolled after introduction of 7-valent pneumococcal conjugate and booster 23-valent polysaccharide vaccine (2001–2004). Source populations were the same for both cohorts. Detailed examinations including tympanometry, video-recorded pneumatic otoscopy and collection of discharge from tympanic membrane perforations, were performed as soon as possible after birth and then at regular intervals until 24 months of life. Analyses (survival, point prevalence and incidence) were adjusted for confounding factors and repeated measures with sensitivity analyses of differential follow-up.

**Results:**

Ninety-seven vaccinees and 51 comparison participants were enrolled. By age 6 months, 96% (81/84) of vaccinees and 100% (41/41) of comparison subjects experienced otitis media with effusion (OME), and by 12 months 89% and 88% experienced acute otitis media (AOM), 34% and 35% experienced tympanic membrane perforation (TMP) and 14% and 23% experienced chronic suppurative otitis media (CSOM). Age at the first episode of OME, AOM, TMP and CSOM was not significantly different between the two groups. Adjusted incidence of AOM (incidence rate ratio: 0.88 [95% confidence interval (CI): 0.69–1.13]) and TMP (incidence rate ratio: 0.63 [0.36–1.11]) was not significantly reduced in vaccinees. Vaccinees experienced less recurrent TMP, 9% (8/95) versus 22% (11/51), (odds ratio: 0.33 [0.11–1.00]).

**Conclusion:**

Results of this study should be interpreted with caution due to potential bias and confounding. It appears that introduction of pneumococcal vaccination among Aboriginal infants was not associated with significant changes in prevalence or age of onset of different OM outcomes or the incidence of AOM or TMP. Vaccinees appeared to experience reduced recurrence of TMP. Ongoing high rates of complicated OM necessitate additional strategies to prevent ear disease in this population.

## Background

Young Aboriginal children in remote Australia have a 24% prevalence of tympanic membrane perforation (TMP) and 15%[1] to 24%[2] prevalence of chronic suppurative otitis media (CSOM). The World Health Organization states that CSOM prevalence greater than 4% indicates a massive public health problem[3].

Among Aboriginal infants, *Haemophilus influenzae*, *Streptococcus pneumoniae*, and *Moraxella catarrhalis *have been isolated from 57%, 34% and 4% of TMPs respectively, with *H. influenzae *and *S. pneumoniae *co-infection in 28% of cases[4]. Pneumococcal serotypes associated with TMP are 19A, 19F, 23F, 14 and 1[5]. The 7-valent pneumococcal conjugate vaccine (7PCV) includes three of these: 19F, 23F and 14 as well as serotypes 4, 6B, 9V and 18C. Trials of 7PCV suggest efficacy of 6% against acute otitis media (AOM) in American and Finnish children[6,7] with greater efficacy against more severe outcomes (9%[7] to 10%[6] against recurrent AOM and 23%[8] to 39%[9] against tympanostomy procedures). Two studies have reported 100% efficacy against TMP due to vaccine serotypes, excluding 19F[8,10]. Efficacy against any TMP or CSOM is unknown. We thought that 7PCV might be even more effective in populations with a high burden of severe otitis (Australian Aboriginal children and others with substantial prevalence of CSOM [11-14]). Pneumococcal vaccination for Aboriginal children in Australia began in late 2001. We compared: 1) time to develop otitis media with effusion (OME) and other OM outcomes, 2) OM prevalence outcomes and 3) OM incidence outcomes in two birth cohorts of Aboriginal infants, before and after introduction of pneumococcal vaccination.

## Methods

### Study Setting

Two cohorts were enrolled from three Aboriginal communities on the Tiwi islands north of Darwin (population 2,029)[15]. We compared a cohort that received pneumococcal vaccination (2001–2004) with a comparison cohort from the same communities prior to vaccine availability (1996–2001).

### Participants

The comparison, or before vaccine group, was enrolled in a randomised controlled trial of long-term amoxicillin versus placebo between 1996 and 2001 (OM-RCT)[16]. As subjects enrolled in the OM-RCT began randomised therapy after detection of the first OME episode, the comparison group for the time to first OME outcome included all subjects enrolled in the OM-RCT. For all other outcomes, the comparison group comprised only those participants assigned to placebo. The vaccinated group was enrolled between 2001 and 2004, after catch-up vaccination for those less than 2 years of age, and introduction of routine pneumococcal vaccination in July 2001.

Comparison group exclusion criteria were: age >12 months, gestation <34 weeks, penicillin sensitivity, long-term antibiotic therapy, craniofacial abnormality, CSOM and immunodeficiency[16]. Exclusion criteria for the vaccinated group were: age >4 months, gestation <34 weeks and congenital abnormality.

### Consent and Ethical Considerations

The mother or carer gave written informed consent for each study. The Institutional Ethics Committee of Territory Health Services and Menzies School of Health Research, and the Tiwi Health Board approved the studies.

### Procedures

Comparison participants were enrolled as soon as possible after birth and examined every two weeks until OME was detected. They were then randomised to amoxicillin or placebo until middle ear status was normal. Children received randomised therapy and monthly examinations for an average of 5.2 months[16]. Vaccinees were also enrolled as soon as possible after birth and examined every two weeks until OME was detected, and then monthly until 12 months of age. Both groups were examined at 12, 18 and 24 months of age or until study completion.

All participants received standard care, including vaccinations, from community clinics. Vaccinees were scheduled to receive 7PCV at 2, 4 and 6 months of age and 23-valent polysaccharide vaccine (23PPV) at 18 months. The research team provided additional clinical care if new problems were identified during assessment. Medications provided by the study and community clinic were supervised for the comparison group but not for vaccinees. Standard antibiotic therapy for AOM among the vaccinated cohort was amoxicillin 50 mg/kg/day for 7 days (research team) and 300 mg twice daily for 5 days (community clinic), and for the comparison group, amoxicillin 50 mg/kg/day for 5 days (research team) and 125 mg thrice daily for 5 days (community clinic).

Assessment involved direct and video-recorded pneumatic otoscopy, tympanometry (GSI 38 Auto-Tymp Grayson-Stadler) and review of clinic records. Two independent, unblinded assessors standardised video diagnoses in the two cohorts. Consensus between assessors' video diagnoses was required in both studies and if a diagnosis in the 1996–2001 cohort was changed. If assessors disagreed a third assessor resolved the decision. Data were recorded using standard forms.

### Definitions

• OME: Type B tympanogram with neutral or mild bulging of the TM.

• AOM: Moderate or marked bulging of the TM. New episodes were defined when the preceding examination was OME or normal.

• TMP: Discharge through a perforation for <6 weeks or pus in the auditory canal. New episodes were defined when the preceding examination was AOM, OME or normal.

• CSOM: TMP with discharge for 6 weeks or greater.

### Microbiology of Tympanic Membrane Perforations

Microbiological techniques were the same for both groups. Aluminium-shafted, cotton-tipped swabs (Disposable Products, Australia) of perforation discharge were collected through, or from as close as possible to, the site of perforation. Specimens were transported and stored frozen as recommended for pneumococcal carriage studies[17]. Aliquots (10 μl) were cultured on selective media and incubated overnight at 37°C in 5% CO_2_. *S. pneumoniae *was confirmed with serotyping by the Quellung reaction, *H. influenzae *by dependence on X and V factors, and *M. catarrhalis *by colony morphology, Gram stain and oxidase production.

### Statistical Analysis

Vaccinee and placebo recipient data were compared, apart from the time to first OME outcome, for which comparison group data were included from all randomised subjects in the OM-RCT; i.e. before detection of OME and subsequent randomisation.

For time to first OME and AOM, data were included if the first examination was before 4 months of age. For time to first TMP and CSOM, data were included if the first examination was before 6 and 9 months of age respectively. Participant data were censored if the duration between consecutive examinations was greater than 2 months. Examinations after 24 months of age were excluded. To explore potential effects of unobserved clinical events because of differential follow-up, sensitivity analyses were performed including different ages at first examination (range less than 4 months to any age) and examination intervals (less than 2 to 6 months).

Analyses of pathogens associated with new perforation (specimen collected <28 days following new perforation) were performed per ear rather than per child. Recurrent (>1 episode in one ear) or bilateral perforation during follow-up were examined by log-linear modelling.

Time to first event outcomes used Kaplan-Meier curves and Cox proportional hazards models. Incidence outcomes used Poisson regression with estimates adjusted for repeated measures using generalised estimating equations. *A priori *analysis included adjustment for 'rate of non-respiratory illness' (incidence of presentations for diarrhoea, anaemia, failure to thrive, skin sores, scabies and fungal infection) as a measure of general ill health between studies. Other potentially confounding variables (gender, age at first examination, rate of well and sick clinic visits, rate of prior antibiotic prescription, birth weight, delayed immunisation (>1 month after due date) were included in forward stepwise modeling if p-values were less than 0.10. Statistical significance was the 5% confidence level. Assuming a baseline median time to OME of 60 days[18], an alpha level of 0.05 with 100 vaccinated and 80 comparison participants, the comparison had power of 0.77 to detect a clinically important difference of 20 days. Assuming a baseline cumulative proportion experiencing TMP of 50%, alpha of 0.05 with 100 vaccinated and 50 comparison participants, the comparison had power of 0.82 to detect an expected reduction of 50% to a cumulative proportion of 25%.

### Role of the Funding Source

The National Health & Medical Research Council of Australia funded the RCT. Wyeth Australia funded the vaccine study. Neither was involved in study design, collection, analysis, interpretation of data, writing or submission of the report.

## Results

### Enrolment and Comparability of Groups

**Figure 1:**
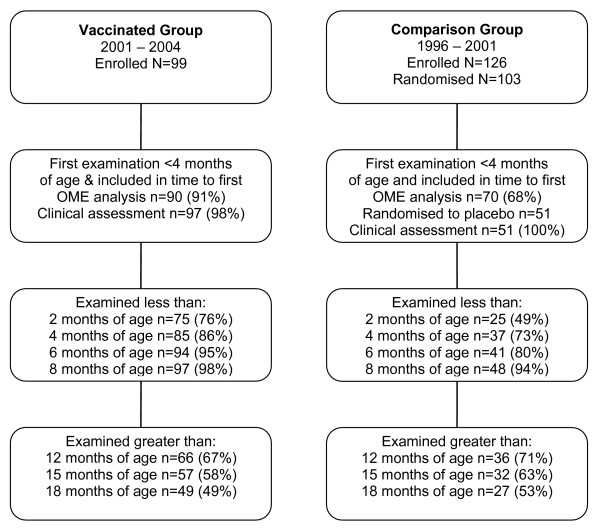
**Enrolment and follow-up profile**. Demonstrating numbers of participants entering and leaving follow-up at different ages.

Greater than 80% of infants in the population after vaccine introduction were enrolled and 54%[16] were enrolled in the before vaccination cohort. There were 97 vaccinees (mean follow-up, 470 days [range 35–728]; Figure 1) and 51 comparison children randomised to placebo (mean follow-up, 472 days [range 154–727]). Mean number of examinations to age 24 months was 10.9 (range 1–22) for vaccinees and 9.5 (range 3–17) for comparison participants. Vaccinees began follow-up at a younger age than comparison participants but by eight months of age similar proportions of each group had begun follow-up (Figure 1). Among vaccinees the third dose of 7PCV was given at a mean age of 6.7 months (range 4.2, 13.6) and at greater than 8 months for 14/93 (15%). Sex distribution, birth weight and maternal age were similar among the comparison groups (Table 1). Compared to comparison participants in the first 6 months of life, vaccinees had reduced rates of lower respiratory illness, were younger at first examination, had fewer well clinic visits, and fewer were late for immunisation (Table 2). Although the reduced rate of lower respiratory illness among vaccinees may be attributable to pneumococcal vaccination, and not potential confounding factors, the other characteristics in Table 2 may still be used for comparison of the two groups.

**Table 1:** Vaccinated and comparison group characteristics

**Participant Characteristic**	**Vaccinated** **N = 97**	**Comparison** **N = 51**	**Mean Difference or Odds Ratio (95% CI)**
Sex (% Male)	45 (46%)	30 (59%)	0.61 (0.29, 1.27)
Birth weight (g)	2956 (n = 87)	3159 (n = 49)	-203 (-409, 3)
Maternal age (years)	24.2 (n = 95)	23.0 (n = 49)	1.2 (-0.8, 3.1)

Birth weight was missing for 10 vaccinees and 2 comparison participants. Maternal age was missing for 2 vaccinees and 2 comparison participants.

**Table 2:** Clinical characteristics of vaccinated and comparison groups in the first 6 months of life

**Clinical Characteristic**		**Vaccinated** **N = 94**	**Comparison** **N = 41**	**Mean Difference or Odds Ratio (95% CI)**
Mean	Age at first examination (in days)	41	60	-18 (-31 – -5)
	Well clinic visits	4.84	10.3	-5.48 (-7.21 – -3.75)
	Sick clinic visits	6.95	8.90	-1.96 (-4.46 – 0.55)
	Upper respiratory illnesses*	1.33	1.00	0.33 (-0.21 – 0.87)
	Lower respiratory illnesses	0.98	3.21	-2.24 (-2.89 – -1.59)
	Non-respiratory illnesses	2.80	2.71	0.09 (-1.06 – 1.24)
	Diarrhoeal illnesses	0.62	0.85	-0.24 (-0.76 – 0.28)
	Antibiotic prescriptions	3.54	4.51	-0.97 (-2.30 – 0.37)

Proportion	Failure to thrive	5 (5%)	1 (2%)	2.25 (0.24 – 109)
	External nasal discharge	27 (29%)	12 (29%)	1.02 (0.45 – 2.31)
	Admitted to hospital	18 (19%)	9 (22%)	0.84 (0.32 – 2.37)
	Late immunisation†	14 (15%)	18 (44%)	0.22 (0.09 – 0.56)
	Tests positive for anaemia‡	24%	36%	-12 (-40 – 15)

Note: 3 vaccinated and 10 comparison participants were excluded from clinical comparison in the first 6 months of life as their first examinations occurred at greater than 6 months of age.

*Upper respiratory illness includes OM or other upper respiratory tract infections.

†Immunisation greater than one month after due date.

‡Anaemia defined as haemoglobin level less than 9 g/dL.

Comparing census data from 2001 and 1996, there were similar income levels, proportions of residents aged 0–4, and rates of unemployment (data not shown). Average household occupancy was lower in 2001 than 1996, 4.5 versus 5.1 occupants per house.[15,19]

### Prevalence of Otitis Media Outcomes in the First Year of Life

By 2 months of age approximately 70% of both vaccinated and comparison participants had developed OME, by 6 months virtually all had developed OME (Table 3). By 12 months, approximately 90% in both groups had documented AOM. The proportion of vaccinees experiencing TMP in the first 9 months of life was lower than comparison participants, although by 12 months 35% of both groups had experienced TMP. By age 12 months, 14% of vaccinees and 23% of comparison participants had developed CSOM (Table 3). Seven children excluded from the OM-RCT due to a diagnosis of CSOM at initial assessment[16] were not included in this study, although their exclusion may have reduced the proportion of comparison participants at risk of developing CSOM.

**Table 3:** Cumulative proportions of participants over time with different otitis outcomes

**Outcome**	**Age** **(months)**	**Vaccinated** **n/N (%)**	**Comparison** **n/N (%)**
OME	1	27/47 (57)	6/13 (46)
	2	49/73 (67)	18/25 (72)
	4	72/83 (87)	35/37 (95)
	6	81/84 (96)	41/41 (100)
			
AOM	2	5/73 (7)	4/25 (16)
	4	33/83 (40)	19/37 (51)
	6	53/84 (63)	33/41 (80)
	9	65/83 (78)	41/45 (91)
	12	65/73 (89)	35/40 (88)
			
TMP	4	3/83 (4)	2/37 (5)
	6	10/84 (12)	8/41 (20)
	9	18/83 (22)	14/45 (31)
	12	25/73 (34)	14/40 (35)
	15	20/59 (38)	14/35 (40)
	24	15/30 (50)	12/23 (52)
			
CSOM	6	1/84 (1)	3/41 (7)
	*9	4/83 (5)	8/45 (18)
	12	10/73 (14)	9/40 (23)
	15	8/59 (14)	7/35 (20)
	24	9/30 (30)	9/23 (39)

To minimise loss of case ascertainment the denominator includes only participants followed to the age of the given category.

*p < 0.05

### Time to First Otitis Media Outcomes

**Figure 2:**
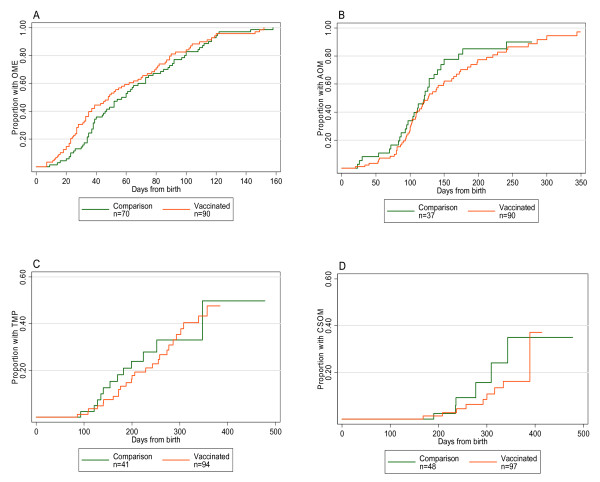
**Kaplan-Meier curves for time to first event**. Time to first: OME (A), AOM (B), TMP (C) and CSOM (D).

Time to first OME analysis included 90 vaccinees and 70 comparison participants. Univariate and multivariate analyses showed time to first OME was not significantly different by group (Table 4). Increased time to first OME was associated with older age at first examination. Univariate and multivariate analyses indicated that vaccination had no effect on time to first AOM, TMP or CSOM (Table 4). Kaplan-Meier curves were similar for both groups (Figure 2).

**Table 4:** Univariate and multivariate survival analyses of time to first event for different otitis outcomes, hazard ratios and 95% confidence intervals

**Variable/s included in analyses**	**OME**	**AOM**	**TMP**	**CSOM**
	
	**Hazard ratio** **(95% CI)**	**Hazard ratio** **(95% CI)**	**Hazard ratio** **(95% CI)**	**Hazard ratio** **(95% CI)**
Univariate				
				
Vaccinated versus comparison group	1.17 (0.85 – 1.62)	0.80 (0.51 – 1.26)	0.84 (0.42 – 1.67)	0.57 (0.20 – 1.62)
Age at first examination (days)	0.98 (0.98 – 0.99)	1.00 (0.99 – 1.01)	n/a	n/a
Females/Males	0.79 (0.57 – 1.10)	1.06 (0.70 – 1.60)	0.99 (0.53 – 1.88)	0.36 (0.11 – 1.13)
Rate of non-respiratory illness*	1.09 (0.79 – 1.52)	1.08 (0.73 – 1.60)	1.07 (0.57 – 2.01)	0.98 (0.39 – 2.46)
Rate of prior antibiotic prescription*	0.71 (0.48 – 1.03)	0.91 (0.62 – 1.35)	0.95 (0.53 – 1.72)	1.63 (0.75 – 3.58)
Birth weight (kg)	0.99 (0.74 – 1.33)	1.04 (0.71 – 1.54)	0.90 (0.49 – 1.66)	0.94 (0.35 – 2.54)
Rate of well clinic visits*	1.10 (0.99 – 1.23)	1.32 (1.07 – 1.62)	1.09 (0.74 – 1.62)	1.10 (0.57 – 2.12)
Late immunisation	0.64 (0.36 – 1.17)	0.69 (0.39 – 1.22)	0.66 (0.31 – 1.40)	1.19 (0.40 – 3.54)
				
Multivariate				
				
Vaccinated versus comparison group	0.90 (0.64 – 1.25)	1.01 (0.61 – 1.67)	0.83 (0.41 – 1.66)	0.56 (0.19 – 1.62)
Age at first examination (days)	0.98 (0.97 – 0.99)	n/a	n/a	n/a
Rate of non-respiratory illness*	1.14 (0.83 – 1.56)	1.04 (0.71 – 1.52)	1.09 (0.58 – 2.07)	1.04 (0.42 – 2.60)
Rate of well clinic visits*	n/a	1.32 (1.05 – 1.65)	n/a	n/a

Note: 150 episodes of first OME in 160 participants, 91 episodes of first AOM in 127 participants, 38 episodes of first TMP in 135 participants, 15 episodes of first CSOM in 145 participants.

* (per month).

n/a not applicable.

### Unadjusted, Multivariate, and Sensitivity Analyses of the Incidence of Acute Otitis Media and Tympanic Membrane Perforation

Analysis of AOM incidence included 90 vaccinees and 37 comparison participants. Unadjusted analysis showed vaccination associated with an AOM absolute rate reduction (ARR) of 0.18 episodes per person-year (2.05 in comparison and 1.87 in vaccinated participants; incidence rate ratio (IRR): 0.91 [95% CI: 0.64–1.29]). Increased AOM incidence was associated with the *a priori *adjustment variable, non-respiratory illness rate, which was included in multivariate modelling (data not shown). Vaccination was associated with an adjusted ARR of 0.20 episodes per person-year (2.03 in comparison and 1.83 in vaccinated participants; IRR: 0.88 [95% CI: 0.69–1.13]). Sensitivity analysis including any age at first examination and less than 4 month examination intervals (97 vaccinees, 51 comparison participants) resulted in a different adjusted ARR of 0.40 episodes per person-year (2.02 in comparison and 1.62 in vaccinated participants; IRR: 0.81 [95% CI 0.67–0.99]). Recurrent AOM (>1 episode) occurred in 45% (44/97) of vaccinees and 55% (28/51) of comparison participants (Table 5).

**Table 5:** Incidence of AOM and TMP in vaccinated and comparison groups, including sensitivity analyses

	**AOM**				**TMP**			
	
	**Baseline analysis**		**Sensitivity analysis**		**Baseline analysis**		**Sensitivity analysis**	
	
	**Vaccinated**	**Comparison**	**Vaccinated**	**Comparison**	**Vaccinated**	**Comparison**	**Vaccinated**	**Comparison**
	
No. of participants	90	37	97	51	94	41	97	51
Examinations per year	11.53	11.80	10.24	10.20	10.77	11.70	10.03	10.20
No. of episodes	104	45	139	87	35	23	37	33
No. with 0 episodes (%)	29 (32)	9 (24)	24 (25)	6 (12)	70 (74)	30 (73)	71 (73)	34 (67)
No. with 1 episode (%)	28 (31)	14 (38)	28 (29)	17 (33)	16 (17)	3 (7)	18 (19)	6 (12)
No. with >1 episodes (%)	33 (37)	14 (38)	44 (45)	28 (55)	8 (8)	8 (19)	8 (8)	11 (22)
Unadjusted incidence	1.87/yr	2.05/yr	1.68/yr	2.02/yr	0.55/yr	0.82/yr	0.44/yr	0.77/yr
Adjusted incidence*	1.83/yr	2.03/yr	1.62/yr	2.02/yr	0.47/yr	0.75/yr	0.39/yr	0.75/yr
Absolute rate reduction^†^	-0.20/yr		-.40/yr		-0.28/yr		-0.36/yr	

* Adjusted in multivariate poisson model considering repeated events.

^† ^Absolute rate reduction is the incidence in the vaccinated minus incidence in the comparison group.

Baseline analyses of incidence of AOM and TMP included participants if the first examination was at less than 4 months and less than 6 months respectively, and included follow-up data if repeated examinations were less than 2 months apart. Sensitivity analyses included participants with any age at first examination, and included follow-up data if repeated examinations were less than 4 months apart.

Analysis of the incidence of new perforation included 94 vaccinees and 41 comparison participants. Unadjusted analysis showed vaccination associated with an ARR of new perforation of 0.27 episodes per person-year (0.82 in comparison and 0.55 in vaccinated participants; IRR: 0.68 [95% CI 0.40–1.14]). Increased incidence of new perforation was associated with increased antibiotic prescription and non-respiratory illness rate (data not shown). Multivariate modelling showed vaccination associated with an adjusted ARR of 0.28 episodes per person-year (0.75 in comparison and 0.47 in vaccinated participants; IRR: 0.65 [95% CI: 0.38–1.10]). Sensitivity analysis including any age at first examination and less than 4 month examination intervals (97 vaccinees, 51 comparison participants) resulted in a different adjusted ARR of 0.36 episodes per person-year (0.75 in comparison and 0.39 in vaccinated participants; IRR: 0.51 [95% CI: 0.31–0.84]). Recurrent perforation (>1 episode) occurred in 8% (8/97) of vaccinees and 22% (11/51) of comparison participants (odds ratio: 0.33 [95% CI: 0.11–1.00]) (Table 5).

**Figure 3:**
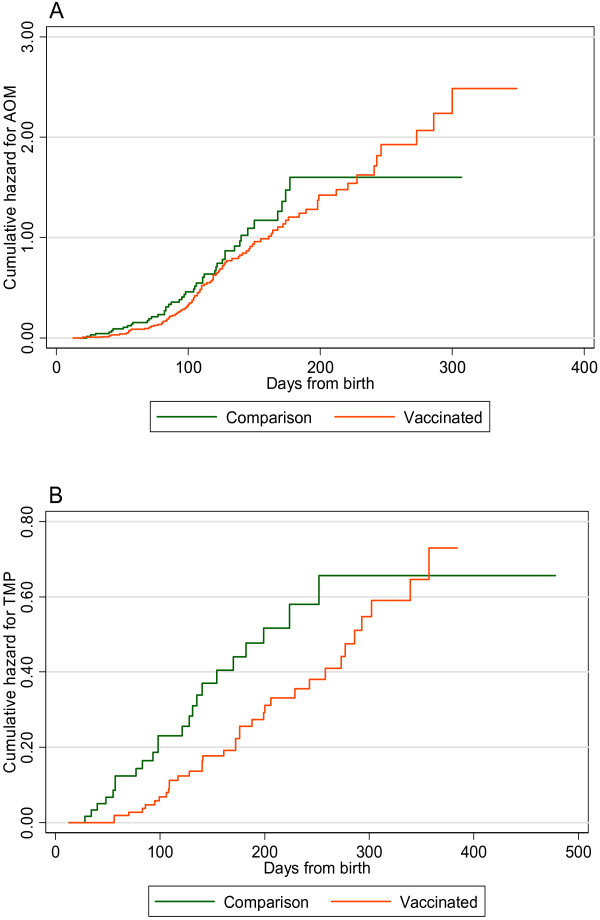
**Cumulative hazard curves for events of AOM (A) and new TMP (B)**.

A lower risk in vaccinees of new perforation, but not AOM, persisted throughout approximately 300 days of follow-up (Figure 3).

### Bilateral and Recurrent Tympanic Membrane Perforation

The number of participants experiencing unilateral or bilateral perforation and single or multiple episodes of perforation were analysed by log-linear modelling. Odds of both left and right TMP during follow-up in vaccinees were 0.41 [95% CI: 0.18–0.94) compared to comparison participants. Similarly, vaccinees had lower odds of multiple perforations 0.40 [95% CI: 0.18–0.87).

### Microbiology Outcomes

Discharge from new perforations was collected more often from vaccinees than comparison participants (Table 6). Proportions of positive specimens (*S. pneumoniae*, *H. influenzae *or *M. catarrhalis*) were similar in the two groups. Proportions of positive specimens with serotypes included in the 7PCV and serotype 6B were lower in vaccinees (Table 6). Predominant serotypes in the comparison group were 6B (5 cases), 23F, 19A, 16F and 11A (2 each). In the vaccinated group 19A, 19F and 16F were predominant (4 each), followed by 6B, 6A and 7C (2 each). In the vaccinated group, vaccine-type pneumococci were associated with 7 episodes of perforation which were distributed evenly throughout the duration of the study (data not shown).

**Table 6:** Pathogens isolated from new tympanic membrane perforations

**Perforation pathogen category**	**Vaccinated** **N = 97 (194 ears)**	**Comparison** **N = 51 (102 ears)**
New perforations	73 (38% of ears)	72 (71% of ears)
New perforation discharge specimen collected	59/73 (81% new perforations)	44/72 (61% new perforations)
Positive discharge specimens*	32/59 (54% specimens)	25/44 (57% specimens)
*S. pneumoniae*	19/32 (59% positive specimens)	16/25 (64% positive specimens)
All vaccine-types	7 (22%)	8 (32%)
Serotype 6B	2 (3%)	5 (20%)
All vaccine-related types	6 (19%)	2 (8%)
Serotype 19A	4 (13%)	2 (8%)
All non-vaccine types	9 (28%)	6 (24%)
Serotype 16F	4 (13%)	2 (8%)
*H. influenzae*	28 (88%)	21 (84%)
*M. catarrhalis*	0 (0%)	2 (8%)
*S. pneumoniae & H. influenzae*^†^	15 (47%)	12 (48%)

* Isolation of *S. pneumoniae*, *H. influenzae *or *M. catarrhalis*.

^† ^Cases of co-infections of *H. influenzae *and *S. pneumoniae *have also been included in the numbers for individual pathogens.

Other serotypes isolated: vaccinated group 19F (3), 6A (2), 7C (2), 23F, 3, 7F, 1 & 19F co-isolated. comparison group 23F (2), 11A (2), 9V, 7F, 13.

## Discussion

This is the first report of the impact of pneumococcal vaccination on any TMP and the natural history of OM in a high risk population. Introduction of vaccination was not associated with large effects on the outcomes of primary interest, delayed onset of OME and proportions of participants experiencing TMP. We also found that infant pneumococcal vaccination in remote Aboriginal communities was associated with, albeit with marginal significance:

a) Little effect on cumulative proportions of participants experiencing, or time to first episode of OME, AOM, TMP, or CSOM.

b) A reduced proportion developing CSOM at 9 months of age.

c) Trends towards reduced incidence of AOM and TMP in the first 2 years of life.

d) Reduced recurrence of TMP.

In addition, the consistent relationship of cumulative hazards for TMP between the groups, suggested that any effect of three doses of 7PCV did not wane before 12 months of age.

The lack of substantial benefit of pneumococcal vaccination for OM in this population is likely due to a combination of factors. Vaccination had little effect on the high prevalence of *H. influenzae *infection or co-infections of *H. influenzae *and *S. pneumoniae*. As with studies in Finland and the US, vaccination did not prevent otitis associated with serotypes 19F[7,8] and 19A[7] which were common in our study. Unlike Finnish[7] and Czech Republic[20] studies of AOM, our data indicate a trend towards increased risk of TMP associated with vaccine-related serotypes. The US also reports increased proportions of AOM associated with vaccine-related and non-vaccine serotypes in the post-7PCV era[21,22]. Increased invasive 19A disease has been noted in post-7PCV surveillance among Alaska native[23] and Massachusetts children[24]. Although our findings do not support substantial replacement OM disease due to *H. influenzae*, they are consistent with US reports documenting potential *H. influenzae *replacement disease following 7PCV[22,25,26]. Limited serotype coverage of 7PCV (50% of comparison group perforations) also contributed to poor vaccine effectiveness. Finally, early age of onset (90% experienced OME and 50% experienced AOM by age 4 months; before the second dose of 7PCV) largely precludes an immunological response to vaccination affecting development of OM. Nonetheless, our finding that vaccinated children had fewer recurrent episodes of TMP suggests that a schedule stimulating an immune response before ear disease is established, e.g. maternal, neonatal or young infant dosing, may be more effective than a 2, 4, 6 month schedule. Although our study did not follow children for a sufficient duration to examine the effect of the 23PPV booster, immunogenicity has been demonstrated[27] with the potential to widen the serotype coverage beyond the 7 serotypes included in the 7PCV. Earlier administration of the booster may be more effective than the 18 month dose although it remains unknown whether boosting with the 7PCV or 23PPV is more effective.

Our study was designed to detect expected large effects. Our results, are however, consistent with more modest effects which would still be important in this population. A further limitation is the use of historic comparisons, which raises the possibility of bias or confounding. Universal vaccination precluded the use of concurrent controls. Although there are limitations, the study setting and measures we have taken, minimise the limitations. Bias due to temporal changes is limited as historic data (1996–2001) are continuous with data collected after vaccine introduction (2001–2004). Ecological data suggested reduced household crowding over time but no change in levels of income, unemployment or the proportion of young children in the population. Bias towards a positive vaccine effect due to temporal improvements in child care practices, health services (e.g. more intensive antibiotic schedules) and environmental conditions is possible, however this was not evident from clinical characteristics before 6 months of age (Tables 1 &2), nor from the essentially non-significant effect that was observed. Potential confounding is reduced as otitis risk factors are universal in this population: breast feeding, absence of pacifiers, family history of otitis and extremely high rates of smoking. Study procedures were objective and held constant throughout the study period. We partially adjusted for potential bias of historic comparisons by adjusting for rates of non-respiratory illness as a surrogate measure of general ill health. Enrolment of 80% of the population in the vaccinated group and 54% in the comparison group, who were also involved in OM-RCT with more restrictive inclusion criteria and supervised therapy, introduced selection bias against a positive vaccine effect.

## Conclusion

Due to potential confounding and bias, the results of this study should be interpreted with caution. Despite the association of infant pneumococcal vaccination with possible reductions in incidence of AOM and TMP, the cumulative proportion of Aboriginal children experiencing OME, AOM, TMP and CSOM was unchanged as was the time to development of different OM outcomes. Of note, the lack of vaccine effectiveness to substantially reduce proportions of participants experiencing different OM outcomes and delayed onset of OM does not imply evidence of no effect. This vaccination is unlikely to reduce the prevalence of ear disease in this population in the first years of life. Our findings have important implications for the generalisability of research conducted in affluent populations for high-risk disadvantaged populations. This assertion is supported by data from Navajo and White Mountain Apache infants, where 7PCV efficacy against clinically-diagnosed and severe OM was somewhat lower[10] than in Californian[6] and Finnish[7] children. Results of post-7PCV licensure studies among affluent populations regarding OM[26,28,29], pneumonia[30] and invasive disease[31] have been very positive. However, 7PCV may have little effect against simple and suppurative ear disease and pneumonia[32] in populations at very high risk of pneumococcal infection and those living in disadvantaged conditions which attribute such risk. Further study of infant cohorts is necessary to document longer term vaccine effectiveness which incorporates potentially increasing indirect effects over time. Thus, the results of efficacy studies and licensing and use of a 10-valent pneumococcal-*Haemophilus *protein D conjugate vaccine, similar to a product shown effective against OM in Europe[20], and a 13-valent PCV[33] are much anticipated. Post-introduction studies of sufficient size, will be needed to better define the impact of these vaccines against complicated OM in the Aboriginal population. Apart from wider serotype coverage (particularly serotypes 19A, 6A and 16F), other potential avenues to improve the effectiveness of pneumococcal vaccines in this population include: maternal, neonatal or young infant vaccination and development of common pneumococcal antigen vaccines. Vaccines with efficacy against OM due to multiple pathogens are also needed. In the meantime, there should be ongoing focus on early detection and treatment of ear disease and hearing impairment, and the underlying, predominantly social and economic, determinants of ear infections in Aboriginal Australians.

## Competing interests

The authors declare that they have no competing interests.

## Authors' contributions

GM joined the study in February 2002, and participated in field work, data collection and entry as well as developing the analysis plan, performing data analysis and writing the manuscript with input from JC, PM and AL. JC assisted in analysis and provided input on the writing of the manuscript. AL developed the study protocol, wrote the funding grant applications, supervised field and laboratory work, assisted in analysis and provided input on the writing of the manuscript. PM developed the study protocol, wrote the funding grant applications, supervised field work, assisted in analysis and provided input on the writing of the manuscript. All authors read and approved the final manuscript.

## Pre-publication history

The pre-publication history for this paper can be accessed here:


